# The lecturer-tutor in undergraduate medical education; navigating complexity as “a recruiter, a timetabler, an administrator, a counsellor”

**DOI:** 10.1186/s12909-023-04560-2

**Published:** 2023-08-15

**Authors:** Enda O’Connor, Evin Doyle

**Affiliations:** 1 Trinity College Dublin, Dublin, Ireland; 2 Department of Anaesthesia and Intensive Care St.James’s Hospital, Trinity College Dublin, Dublin, Ireland

**Keywords:** Undergraduate medical education, University tutor, Complexity theory, Professional identity

## Abstract

**Background:**

Tutors play an important role in the delivery of effective undergraduate medical education (UGME). These roles commonly involve competing clinical, educational and research commitments. We sought to obtain a rich description of these posts from doctors working in them.

**Methods:**

We used a pragmatist, sequential explanatory mixed-methods design with a sampling frame of clinical lecturer/tutors in 5 Irish medical schools. Purposive sampling was used for recruitment. Quantitative data collected from a validated online questionnaire were used to inform a semi-structured interview question guide. Thematic analysis was conducted independently by each of the study researchers, using a coding frame derived in part from the findings of the online questionnaire. Quantitative and qualitative mixing occurred during data collection, analysis and reporting.

**Results:**

34 tutors completed the online survey with 7 volunteers for interview. Most respondents took the job to gain experience in either educational practice (79.4%) or in research (61.8%). Major themes to emerge were the diverse interactions with students, balancing multiple professional commitments, a high degree of role-autonomy, mis-perception of role by non-tutor colleagues, challenges around work-life balance and unpredictable work demands. Using a complexity theory lens, the tutor role was defined by its relational interactions with numerous stakeholders, all in the context of an environment that changed regularly and in an unpredictable manner.

**Conclusions:**

The undergraduate tutor works in a demanding role balancing educational and non-educational commitments with suboptimal senior guidance and feedback. The role is notable for its position within a complex adaptive system. An understanding of the system’s interactions recognises the non-linearity of the role. Using a complex systems lens, we propose improvements to undergraduate education centred around the tutor.

**Supplementary Information:**

The online version contains supplementary material available at 10.1186/s12909-023-04560-2.

## Background

Universities and medical schools are tasked with the responsibility for designing and delivering effective medical education to ensure that graduating students have acquired the knowledge, skills and attitudes to commence safe and effective clinical practice. Notwithstanding innovative approaches to undergraduate medical education (UGME) such as problem-based learning [1] and technology-enhanced learning [2], there is an ongoing requirement, to varying degrees, for teaching to be delivered to students. And for this to occur, educators are needed.

Undergraduate teaching evaluation questionnaires highlight the importance of educators in the learning process. In the 25-item UCEEM questionnaire, 9 items (36%) directly relate to the staff involved in the delivery of student teaching [3]. Eleven (22%) of 50 DREEM items relate to teachers [4]. All 14 items on the Maastricht Clinical Teaching Questionnaire evaluate the teacher [5]. In the current Times Higher Education (THE) ratings, 19.5% of the rating is awarded for teaching reputation and ratio of students to teaching staff [6].

The World Federation for Medical Education (WFME) provides guidance for medical schools on maintaining quality in UGME. In particular, they note that “adequate numbers of well-trained and committed academic staff (also referred to as faculty or teachers), supported by technical and administrative staff, are critical to the effective delivery of the curriculum.” [7] (p.22). Furthermore, the WFME specify that medical schools “develop a clear statement describing the responsibilities of academic staff for teaching, research, and service” [7] (p.23) and ensure that academic staff have appropriate information, induction and preparation to enable delivery of the teaching curriculum.

In an international context, there are geographical differences in third-level faculty terminology; undergraduate medical educators are denoted by terms such as clinical academic, teacher, coach, lecturer, tutor, facilitator or demonstrator. Some of these are full-time education roles funded by a parent university. Others are consultant-grade doctors in university-affiliated hospitals occupying titled university positions delivering a combination of clinical care and undergraduate education. A third group are non-consultant doctors with temporary university tutor appointments, who have a combination of clinical, educational and research duties. These non-consultant appointees are an important pillar supporting the delivery of UGME in jurisdictions including the UK, South Africa, Canada, Australia and Ireland [8–13]. Some countries advocate for clinical academic pathways, where postgraduate medical training includes formal training in research and/or education [10, 14, 15].

Recent published literature has highlighted issues around posts which combine clinical and academic duties. While they broaden skills outside the clinical sphere [16] and help crystallise career goals [8], clinical academic/tutor roles have their attendant problems. By definition, they require a commitment to more than one manager, described in one study as akin to “riding two horses” [17] (p.2). Frequently, clinical commitments tend to crowd out research and educational duties [8]. These posts are busy jobs which spill over into life outside work and carry a risk of staff burnout [9, 17]. A lack of clarity over role definition and expected duties is not uncommon [18] as is a poor understanding of the job by non-academic colleagues [18]. Notwithstanding these insights however, most of these published insights stem from nursing research and from jurisdictions with formal clinical academic training pathways.

In contrast, most of the undergraduate medical tutors in Irish medical schools are doctors who take a temporary break from full-time clinical practice to occupy standalone full-time university-funded posts, usually for a period of 12 months. All posts combine the three domains of clinical, research and educational work, and although there are institutional differences, job descriptions specify that a majority of time (50–60%) is spent doing educational activities. Clinical and research duties comprise approximately 20% of work time respectively. All of these tutor posts are filled at competitive interview. They represent a key pillar of undergraduate medical teaching, bridging as they do universities, medical students and the clinical environments in which undergraduate learning occurs. No prior studies have explored their experience of their role in UGME.

Accordingly, in light of existing research around undergraduate clinical academics, but the relative paucity of studies of medical tutors involved in UGME, we designed a study to gain an understanding of the undergraduate medical tutor post from the perspective of people working in that role. Despite the varied structure of UGME across different countries and medical schools, we predicted that our findings would resonate internationally with those involved in the organisation and delivery of undergraduate clinical teaching, and in particular those managing a combination of educational, clinical and research commitments. The purposes therefore of the study were to explore the tutors’ experiences of the job and the duties involved, to elucidate a rich description of the role, to highlight factors which might mitigate some of the concerns reported by comparable educators in existing studies in this area and inform improvements in medical student learning.

## Methods

### Design

Taking a pragmatist approach to the research problem, a mixed-methods design was selected for the study. This was chosen as the best method for understanding the complex, multifaceted role of the clinical tutor working in diverse environments with commitments to multiple individuals [19]. Specifically, the purposes of combining quantitative and qualitative data were complementarity (enhancing and elaborating on one dataset with a second) and development (using the quantitative data to inform the design of the qualitative data collection strategy) [20]. Mixing occurred at the stages of data collection, analysis and reporting, the latter through textual description of the integrated findings [21]. In reporting the results, equal emphasis was placed on the quantitative and qualitative research strands.

#### Ethics approval

for the study was obtained from the School of Medicine Research Ethics Committee, Trinity College, Dublin.

### Setting and participants

Of the six medical schools in Ireland, five employed educational staff in a capacity which matched the description of a clinical tutor (a non-consultant doctor with predominantly undergraduate education duties but with additional clinical and research commitments). Purposive sampling was used, with the sampling frame being the total number of clinical tutors working in these five medical schools. A link to an online survey was sent to the clinical tutors by a third party not involved in the study (a member of administrative staff from each medical school), followed by subsequent email reminders 2-weeks and 4-weeks later. At the end of the survey, participants were invited to participate in an interview to further discuss the tutor role. Volunteers expressed an interest by emailing a third party, a member of secretarial staff in the principal study centre who was not involved in any other part of the research project. These details were forwarded to the researchers, who subsequently made contact with volunteers by email.

### Data collection

The online survey consisted of 31 questions related to the clinical tutor post, combining multiple-choice (MCQ), closed-ended, Likert scale and free-text questions, circulated using a Qualtrics© platform (see Appendix 1). The validity of the survey tool was optimised through an iterative design process involving consultation with two clinical tutors who were not otherwise involved in the study. These stakeholder consultations enhanced (a) content validity by ensuring that key aspects of the clinical tutor role were included within the scope of the survey questions, and (b) face validity, strengthening the link between the information in the survey answers and that which the authors sought to measure.

Interviews were conducted online by one of the researchers (ED) using videoconferencing. In keeping with sequential explanatory mixed-methods research, the question frame for the qualitative structured interviews was informed by the findings from the quantitative online survey questions. There were 13 interview questions in total; 10 addressed concepts drawn from the survey (see Appendix 1). Two further questions sought a general overview of the post and any suggestions about how it might be improved. The last question was open-ended to allow participants to raise any previously unaddressed issues, thereby enabling the emergence of any unanticipated data. The principal purpose of the interviews was to provide an in-depth explanation for some of the survey findings, in particular related to the following aspects of the tutor post; balancing competing professional commitments, institutional support and feedback, job description and structure, non-educational duties, and perceived self-identity.

### Data analysis

The quantitative results were analysed using descriptive statistics on Microsoft Excel©. This was a collaborative process done together by the two authors.

Interview transcripts were analysed using coding reliability thematic analysis [22]. Deductive analysis was the predominant strategy, using a coding frame derived in part from the results of the quantitative survey but also from the authors’ experience in this area of research and from consultation with clinical tutors during validity assessment of the survey. These predefined codes centred around tutors’ perceived identity, role commitments, role clarity, role expectations and institutional support. Inductive coding was also used, whereby the authors sought to identify any topics not accounted for in the predefined coding frame. Interview coding was carried out separately by the two authors. Through a series of subsequent meetings, any differences in findings, in particular those related to topics outside the coding frame, were discussed and agreed prior to the final thematic summary. Participants were invited to view the transcripts of their interviews to fact-check the content and to clarify and to recommend corrections if required. None opted to view the transcripts.

### Study rigour

For the quantitative part of the study, the validity of the survey tool was enhanced by a process of survey piloting. Furthermore, reliability was evaluated by calculating Cronbach’s alpha for 5 variables (involving 7 survey items). These 5 values were 0.45, 0.58, 0.73, 0.78 and 0.90 (See Appendix 2). From a qualitative perspective, credibility, transferability and dependability were enhanced by the use of source triangulation, participant quotes, verbatim transcripts, member checking, thick description and purposive sampling [23]. The 6-point GRAMMS guidelines were followed to strengthen the overall rigour from a mixed-methods standpoint [24]. Accordingly, we have outlined the justification for choosing a mixed-methods strategy, the details of the explanatory design, the details of each research strand, the timing and process of integration, and the insights of selecting a mixed-methods approach [25].

## Results

Thirty-four tutors (of a total of 138) from 5 Irish universities responded to the online survey (S1-S34), 61.8% of whom (21 respondents) were female. This was a response rate of 24.6%. Seven tutors (P1-P7) volunteered to participate in video interviews, which lasted for 23–56 minutes. Most of the tutors worked in the specialities of medicine (13; 38.2%), surgery (9; 26.5%) and paediatrics (5; 14.8%). Twenty-three tutors (67.6%) were pursuing a postgraduate qualification (PhD, MD, MSc, Postgraduate Diploma) and 13 (38.2%) were studying for an upcoming postgraduate examination. The most common reasons for taking the tutor role were to gain experience in teaching and education (27; 79.4%) and to do research (21; 61.8%). While 17 (50%) of respondents had prior teaching experience, only 6 (17.6%) had a formal qualification in medical education (Certificate, Diploma or Masters). A majority of respondents (25; 73.5%) were following a career path towards hospital consultant practice and a similar number (26; 76.5%) wanted a direct involvement in medical education in their future career. Career aspirations in research were less commonly reported (14; 41.2%).

### Description of post

The job combined a high workload with a high degree of job satisfaction. It was described as “a very tough post”(P6), “a challenge and a struggle”(P7), “workload is massive”(S21), “more time-consuming than expected”(S34), a “hectic lifestyle”(S13). One interviewee reported; “I think the job entails a lot more than what people think”(P6). Numerous references were also made to the positive perceptions of the post; “excellent”(S27), “very rewarding: (P1), “very satisfying”(S12,S7), “really enjoyed the job”(S28), “a largely positive experience”(S14), “a rewarding thing to do”(P4). The frequency of role activities (clinical, educational, research and administrative) is shown in Table 1. The perceived benefits of the job are shown in Table 2.

**Table 1:** Activities related to the tutor role and how frequently the respondents reported that they occurred at least once weekly (in the survey, respondents were asked whether activities occurred less than once weekly, 1–3 times weekly or > 3 times weekly)

Educational activity	Occurring at least once weekly no. of respondents (%)
Small group face-to-face teaching/bedside tutorials	25 (73.5)
Online tutorials	20 (58.8)
Large group lectures	14 (41.2)
Student assessment	11 (32.4)
Simulation teaching for students	6 (17.6)
Teaching non-student healthcare staff	3 (8.8)
**Non-educational activity**	**Occurring at least once weekly**
Clinical work during daytime hours	21 (61.8)
Clinical research activities	14 (41.2)
Organisation/Administrative duties	13 (38.2)
Discussions with university supervisor	7 (20.6)
Clinical work on-call shifts	4 (11.8)
Laboratory research activities	3 (8.8)
**Feedback-related event**	**Occurring at least once weekly**
Informal feedback from students on teaching	4 (11.8)
Informal feedback from supervisor on teaching	2 (5.9)
Formal written feedback from students	0 (0)
Formal written feedback from supervisor	0 (0)

**Table 2:** Responses to survey questions about the outcomes of the tutor post. (Positive = strongly agree/somewhat agree; Negative = strongly disagree/somewhat disagree; Neutral = neither agree nor disagree)

Survey question	Positive	Negative	Neutral	No answer
The job has improved my knowledge and/or skills in medical education	29 (85.3)	1 (2.9)	4 (11.8)	0
The job has improved my knowledge and/or skills in conducting clinical research	17 (50)	7 (20.6)	9 (26.5)	1 (2.9)
The job has improved my knowledge/skills in conducting lab-based research	8 (23.5)	18 (52.9)	7 (20.6)	1 (2.9)
The job has improved my knowledge and/or skills in the linked clinical specialty	25 (73.5)	6 (17.6)	1 (2.9)	2 (5.9)
This job has made me more likely to consider medical education as a major component of my future career	27 (79.4)	5 (14.7)	1 (2.9)	1 (2.9)
The job has provided me with knowledge and/or skills that will be useful for me during my career	30 (88.2)	0	2 (5.9)	2 (5.9)

Integration of the quantitative and qualitative data yielded six overarching themes as follows:

Educational and non-educational nature of interactions with students.

Balancing multiple roles with limited guidance.

Role autonomy.

Suboptimal research opportunities.

Perception by non-tutor colleagues.

Paucity of feedback on tutor performance.

### Educational and non-educational nature of student interactions

Teaching students was reported to be the most enjoyable aspect of the job. (32 of 34 survey free-text answers). The phrases used to describe these pedagogical interactions [supporting, mentoring, listening, meeting, “seeing them progress and improve”(S14), “seeing students learn”(S30), “helping them understand medicine”(S1)] suggest a role beyond that of teacher, encompassing one of general support and guidance.

Conversely, interactions with students were frequently challenging, such as with underperforming students, when delivering negative feedback and when mediating between the student and the university administration. These interactions often occurred at short notice, as “students contacting outside of hours”(S12) and in the context of an uncertain governance structure [“There is often confusion over who has the final say on things”(S28)]. Some students, such as those approaching end of year assessments or navigating new clinical environments (e.g. intensive care unit, anaesthetic department) required psychological support that tutors felt ill-equipped to provide [“I wouldn’t be the most appropriate person that they should be talking to”(P7)]. And while all universities have pathways for psychological support and grief-counselling, tutors “definitely have a role in picking up students who are struggling”(P6) and “…decide when they need counselling and further support.”(S18).

### Balancing multiple roles with limited guidance

Survey respondents perceived teaching to be their most important commitment [32(94.1%) extremely or very important], followed by clinical research [16(47.1%)] and clinical work [12(35.3%)]. Nonetheless, tutors balanced numerous commitments in different professional roles, described by two respondents as “a teacher, a helper, a researcher, a doctor”(P7) or “you’re a recruiter, you’re a timetabler, you’re an administrator, you’re a counsellor”(P6). Roles were also geographically dispersed [“sometimes I literally have to drive to three different places on one day”(P7)]. One survey respondent described it as “balancing multiple commitments in different places for different people with everyone thinking their part is the most important”(S11). Twenty survey respondents (58.8%) felt they had duties that would be better done by others; these were predominantly administrative tasks such as booking teaching areas, coordinating learning timetables and recruiting the teaching staff. These tasks added considerably to the overall workload but also to the unpredictable nature of the work [“so much chop and changing happening(P6)”; “I did have to do a lot of admin and moving around timetables and scheduling”(P4)]. Organisational shortcomings led to the need for revisions [“…we had to create a new timetable like ten times in a row”(P5)] and to unexpected increases in educational demands such as “being given far far more students than the department normally has”(S6).

In light of these commitments, some tutors reported a lack of institutional guidance, which would have helped overcome the normal “natural uncertainty”(P4) of the job. Respondents highlighted “a lack of rostered or defined duties”(S20), recommending “a little bit more structure about what was expected of you”(P1) or that “a clear job description would have helped”(P3). One survey respondent “expected more structure, more teaching and less organising and admin work”(S26).

### Role autonomy

In light of the lack of institutional guidance, the finding that much of the daily educational work and delivery of teaching was self-directed is not surprising. Of note however, only half of the tutors had prior teaching experience and less than 1 in 5 had prior formal medical education training. While over three quarters of survey respondents confirmed the presence of an undergraduate curriculum, “largely the content was there but then how you chose to deliver it or what you chose to emphasise was kind of your own”(P4). Some respondents viewed this autonomy as a benefit of the job, helping with leadership training [“scope to innovate”(P3)] and facilitating professional development [“I have learned my own way of doing things”(P6)]. It also promoted a better work-life balance, enabling the “ability to work from home a few days a week”(S29) and “having more autonomy over my schedule”(S17).

### Suboptimal research achievements

Only 14(41.2%) and 3(8.8%) of survey respondents were engaged in clinical and lab-based research respectively on at least a once-weekly basis, notwithstanding that nearly two-thirds applied for the job to gain experience in research practice. Only 17(50%) and 8(23.5%) thought the job positively impacted on their learning in clinical and lab-based research respectively (see Table 2). While regular research activities were very satisfying for a minority of tutors, it was a lesser priority for many others [“most of the time it feels like research can wait another day”(P2)], the implication being that clinical and teaching duties are priorities that cannot wait. This was described by one interviewee as “the research is dependent on you and how much time you’ve left after you’ve contributed towards a clinical and educational part”(P6). Furthermore, achieving a meaningful research outcome within 1 year was noted to be difficult, and served as a demotivating influence.

### Perception by non-tutor colleagues

Despite the large workload and multiple competing roles, one of the greatest challenges was the lack of insight non-tutor staff had about the job, what survey respondents called a “misunderstanding of dual roles”(S19) and a “lack of recognition of the time heavy nature of the role”(S11). Consequently, there was an expectation that tutors were always available to fill workforce gaps [“…if there’s any shortage on the team or there’s someone not in clinic,…people would be like oh yeah, the tutor can cover…”(P7)]. This perception was in part because tutors worked in different sites had a lesser presence in any one clinical site [“we were deemed very invisible”(P4)]. It was also viewed as “selling out” and moving to an easier job [“I think just the general perception of clinical tutors is that people just don’t do much work. They just sit down in the office drinking coffee”(P7)]. The tutors felt they were no longer a legitimate member of the tribe of junior doctors, defined by a shared experience and identity, described as “…the perceived glory of the NCHD (non-consultant hospital doctor) is that you’re overworked and, you know, it’s hard and chaotic…I understand that and there’s a solidarity”(P4). One interviewee described it as “I’ve felt like this outsider…I think that was the hardest bit of the job”(P4).

### Paucity of feedback on tutor performance

In parallel with self-direction was a paucity of direct supervision and feedback about learning. This raised concerns about the quality of education [“I suppose if you want to maintain maybe a sense of best quality and best standards, probably every tutor maybe should be observed at some point by like a senior lecturer”(P4)]. Tutors rarely received feedback on their educational performance. None received formal weekly feedback, and one in eight received informal feedback, most commonly from student learners [“You only really get feedback from students”(P7)]. Formal feedback was sometimes self-directed [“I sought out feedback. If it wasn’t for that, feedback would have been limited”(P3); “…if I didn’t voluntarily sign up for that diploma, I would have had no feedback”(P4)] or based on self-evaluation [“you judge yourself how you feel the sessions are going…”(P4)].

## Discussion

Using a sequential explanatory mixed-methods study design, we have explored in detail the role of the clinical undergraduate tutor in a national context across 5 Irish medical schools. Our findings indicate that tutors work within a system characterised by a high workload, numerous unpredictable commitments, both educational and non-educational, suboptimal structural and feedback support and a general under-recognition of the role by non-tutor colleagues. Nonetheless, tutors reported high levels of job satisfaction, in particular with broadening medical education skills and influencing career direction. Aspirations around research achievements however were largely unrealised. Furthermore, while the tutor role provided a work-life balance not usually afforded to busy non-consultant doctors, this was partly offset by concerns around professional identity.

We anticipated that the use of a mixed-methods design would enhance the richness of the data collection and analysis, and this indeed proved to be the case. In general, the survey enabled the identification of key issues around working in the tutor role whereas the qualitative data collected at interview allowed a deeper exploration of the details around these issues. This was particular evident in our explorations of professional identity, role conflict and autonomy, feedback, research commitments and of the under-appreciation of the role by non-tutor staff. Without the interviews, we would not have gained such research depth in these findings.

Our findings overlap with those in previous research. A recent mixed-methods study exploring the experiences of doctors, nurses and allied health staff pursuing a clinical academic pathway highlighted issues around under-appreciation of the role by non-academic staff and difficulties balancing educational and clinical commitments [9]. Furthermore, they also noted a disparity between prior enthusiasm for research practice and low satisfaction from research achievements once in the role. In our study, the high level of self-directed practice may have undermined the research potential, where a greater amount of direct supervision and guidance is likely to be needed to achieve research outcomes. Newington et al. [18] interviewed 20 nursing clinical academics and managers, noting how respondents’ peers tended to view clinical academia as a “vanity project”(p.385). The burden of administrative duties on academic clinicians is also well described, amounting to as much as 24% of total workload in one report [26]. Role conflict and role ambiguity, both evidence in our findings, are well-recognised in clinical academic communities [9, 17, 27].

Taking a broader view of student learning, UGME could be viewed as a “system”, which, in an educational context implies the presence of inputs, processes and outputs (IPO) [28]. A simple linear system is one in which outputs are proportional to inputs and processes occur sequentially and are independent of each other [29]. A typical example would be a standalone educational course with defined learning objectives, material and methods, delivered by a discrete faculty group and with an assessment tool for measuring outputs. UGME however, from a tutor’s perspective, displays features of a complex system (i.e. “a messy…uncertainty of living systems…where the relationship among things is more than the things themselves” [30] (p.835]). The complexity of the tutor role stems from our ability to view it “…in terms, not of itself, but of its relations” [31]. The job is a nexus between several large entities (the clinical, university and research environments, and the student body) and it is striking how the tutor role was defined, throughout our survey and interviews, by its numerous relational interactions between these entities – with students (individually, in small groups, in lectures), with university staff, with clinical supervisors, with academic supervisors, with research staff and with non-tutor clinical colleagues – all in the context of an environment that regularly and unpredictably changes. Every tutor role is different and each individual tutor role develops and changes dynamically over time, often in response to unpredictable circumstances.

A key aspect of complexity is interconnectedness, the relationships between individual elements of a system. Furthermore, complex systems are said to adapt, or display “emergence”, through the interactions between these elements rather than by the independent actions of any one element [32–34]. Therefore this raises the question about whether a complex system can be studied and understood by researching one component of the system (e.g. the clinical tutor in UGME). Some authors propose that the agency of one individual (or a group of individuals) driving change is compatible with a complex system [35]. Therefore, the role of an individual tutor can be viewed as a change-agent in a complex system of undergraduate education as they contribute to a process of what Mennin calls “co-evolution” [30] (p.837) and what Kehoe describes as a process of disruption (“challenging the status quo”) [17] (p.383). Interestingly, co-evolution was not confined in our study to the tenure of one tutor, but carried on from year to year. As described by one interviewee, “Any information that I really got about how the year ran was from last year’s tutor…and I’m going to pass all that information onto next year’s tutor”(P1) recognising “we put our own twist on it when we take it from other people”(P7).

Conversely, Kannampallil et al. [36] highlight the “non-decomposability” of complex systems, positing that they cannot be adequately studied by focusing on only one individual component of the system. Nonetheless they recognise that complex systems cannot be researched as a whole, that a certain perspective needs to be selected, and that “complex systems can appear very different, depending on the aspects, granularity, and circumstances the researcher chooses to focus on” (p.945). Our study, through a rich mixed-methods approach, recognises the pivotal role the tutor plays in the interconnectedness between the individual components in UGME. Of course, there are relational processes between the university, medical school, students, clinical teachers, clinical doctors, administrators that are independent of the tutor. There are numerous perspectives on which UGME could have been researched but our study views the system from the point of view of the interconnectedness around the undergraduate tutor within the system.

Does the use of a complexity lens help us understand how to better design and deliver tutor-based UGME?

First, we propose that it helps understand the role and its context. To take on the tutor role is to become embedded in a complex system, to understand the presence of unpredictable variables, to recognise the non-linearity of the educational interactions, to be an agent facilitating incremental change and to accept that the duties are at best a combination of proactive and reactive practice within an environment where all variables cannot be controlled.

Second, it enables improvement strategies that draw from published literature. Reed et al. [37] have, in a healthcare setting, proposed a set of rules to translate evidence about complex systems into practice, to provide “actionable guidance to both practice and research” (p3). Though these do not directly relate to an educational setting, many of the rules have immediate relevance to a system of undergraduate medical education. We have applied these rules to the UGME setting, providing suggestions about how practice could be improved if viewed from the perspective of complexity theory (Table 3).

**Table 3:** Proposed strategy to translate complexity research to practice, applied to the clinical tutor role in undergraduate medical education. “Rules” and “Descriptions” are all derived from Reed et al. [37]. UGME: undergraduate medical education

Rule	Description	Example in UGME practice
Understand the problem and the opportunities	“…any intervention is sensitive to the unique initial conditions of the local system” (p9)	- Recognition that the system of UGME in each medical school is unique and requires an individual tailored approach to improve
Identify, test and iteratively develop potential solutions	“…testing intervention ideas in practice and responding to insights and challenges that are often difficult to anticipate” (p10)	- Regular discussions (a) between tutor and university, clinical, research and student representative and (b) within group of tutors, exploring issues and possible solutions. - Formal handover process from 1 tutor to the next
Assess whether improvement is achieved and capture/share learning	Measure outcomes and share with the groups within the system	- Collect data (quantitative and qualitative) from multiple sources (students, tutors, clinical staff, university staff) - Share findings with groups to foster discussion about improvements
Invest in continual improvement	“Translation cannot be seen as a one-off activity and ongoing monitoring and review needs to guide actions to…support long term success” (p11)	- Tutor and clinical staff changeover occurs at the same time, compounding this problem - Formal handover process from 1 tutor to the next - Annual performance review using agreed metrics
Understand practices and processes of care	“…project teams need to look beyond individual competence or actions to understand the complex interactions…that determine quality of care” (p12)	- Orientation of tutors should include reference to and learning about systems, in particular complex systems. This will promote understanding about directing inputs appropriately (addressing other components as well as personal agency)
Understand the type and sources of variation	Gain insight into variables that can and cannot be controlled	- Optimise controllable variables: e.g. availability of faculty and teaching space during lunchtime - Recognise uncontrollable variables: e.g. staff sick-leave, demand from clinical team, student crisis - Consider a UGME “care bundle” to improve system resilience and reduce impact of variation. This would include specific guidance on faculty allocation, curriculum, teaching venues
Identify systemic issues	“Achieving an overall improvement requires many other aspects of the system, and related systems, to be ‘fixed’” (p12)	- Recognise that an increase in tutor’s work and time will not necessarily translate into better student learning - Improve support from administrative staff, teaching faculty, IT staff, university counsellors so more tutor input is targeted to educational output
Seek political, strategic and financial alignment	“…project teams need to build strategic and political alignments with other systems stakeholders to influence areas beyond their control” (p13)	- Tutor not well placed to influence senior stakeholders - Clinical and university supervisors should recognise issues and advocate for appropriate staff and resources for the tutors
Actively engage those responsible for and affected by change	“Frontline staff and patients need to be central in the planning, design and…quality improvement endeavours” (p13)	- Student engagement in activities seeking feedback and suggestions for change and improvement - Mid- and End-of-Term discussions with tutors seeking quality improvement suggestions
Facilitate dialogue	Facilitates “…social sense-making, increasing understanding of each other’s experiences of being in the system, to learn how these can better coexist…” (p14)	- Regular meetings with representatives from all groups (tutors, students, university academic, university administrative, research, clinical) to highlight issues and discuss solutions - Build a community of practice of tutors to share experiences and possible solutions
Build a culture of willingness to learn and freedom to act	“…it is necessary to have the humility to accept that answers cannot be fully known in advance, that learning will occur base on experience” (p14)	- The system of UGME cannot be summarised in one guidance document. Some “learning on the go” is required. - Give system groups the opportunity to regularly report to supervisors about new discoveries based on their experiences
Provide headroom, resources, training and support	“Many of the skills required to understand and intervene in complex systems are not commonly taught to healthcare professionals” (p14)	- Ensure tutors are given the support (administrative, feedback, faculty) they need to effectively do their job - Provide a clear curriculum with learning methods and objectives - Include complex systems learning in tutor orientation

Our study has limitations. The most obvious is the small proportion of total tutors who responded to the survey. Notwithstanding this, all Irish medical schools with tutors were represented in the participants, as were seven of the main clinical disciplines (including psychiatry, anaesthesia/critical care obstetrics/gynaecology and radiology). Moreover, by adopting a mixed-methods approach, qualitative data added richness to the analysis. in particular in the following areas; reasons for poor research engagement; issues around psychological support for students; tribal aspects of professional identity; the why and how of poor formal feedback.

In conclusion, our study views the undergraduate tutor role through the lens of complexity theory. Using this theory, we propose improvements that may enhance the experience of doctors in the role as well as improving the educational outcomes for medical students.

### Electronic supplementary material

Below is the link to the electronic supplementary material.

Appendices: 1. Questionnaires used in survey and semi-structured interviews. 2. Cronbach alpha values for the survey questionnaire

## Data Availability

The datasets used and/or analysed during the current study are available from the corresponding author on reasonable request.
