# Supplementary material for: The lecturer-tutor in undergraduate medical education; navigating complexity as “a recruiter, a timetabler, an administrator, a counsellor”
Source: BMC Med Educ. 2023 Aug 15;23:575. doi: 10.1186/s12909-023-04560-2 (PMC10428569; doi:10.1186/s12909-023-04560-2)
Supplement: Supplementary file 1 — Appendices: 1. Questionnaires used in survey and semi-structured interviews. 2. Cronbach alpha values for the survey questionnaire [file 12909_2023_4560_MOESM1_ESM.docx]

**Appendices:**

Title:

The lecturer-tutor in undergraduate medical education; navigating complexity as a “recruiter,a timetabler, an administrator, a counsellor”

Author information

Enda O’Connor (corresponding author)

Trinity College Dublin, Dublin Ireland

Department of Anaesthesia and Intensive Care, St James’s Hospital, Dublin 8 Ireland

[oconnoen@tcd.ie](mailto:oconnoen@tcd.ie)

ORCID: [0000-0002-9558-6061](https://orcid.org/0000-0002-9558-6061)

Evin Doyle

Trinity College Dublin, Ireland

Department of Anaesthesia and Intensive Care St.James’s Hospital, Dublin8, Ireland

ORCID: [0000-0003-3179-9436](https://orcid.org/0000-0003-3179-9436)

**APPENDIX 1: QUESTIONS FOR SURVEY AND INTERVIEWS**

SURVEY

1. What institution(s) are you currently working in? (please include university and hospital[s])
2. In which clinical department(s) do you current teach AND work? (e.g. surgery, psychiatry, pathology, cardiology etc.)
3. With which gender identity do you most identify?
4. What year did you graduate from medical school?
5. Did you do a graduate-entry medical degree? (if yes, what degree[s] did you possess prior to starting Medicine?)
6. Did you have any training, experience or qualification in medical education before starting this job? If yes, please explain.
7. Why are you working in the tutor/lecturer job? Choose >1 of the following if they apply:
   1. To gain experience in teaching and medical education
   2. To have an opportunity to do research
   3. To gain clinical experience in the associated clinical specialty
   4. It was my only job option
   5. I was uncertain about my career interests
   6. I wanted to stay working in a specific region in Ireland
   7. Other (please explain)
8. Have you had any formal training in medical education since you started this job? If yes, please explain.
9. Are you currently undertaking a masters/PhD programme? If yes, please explain.
10. Are you currently preparing for any postgraduate exams? If yes, please explain.
11. Please describe your current career aspirations (e.g consultant cardiologist, general practitioner, consultant obstetrician).
12. Do your career aspirations include full- or part-time medical education responsibilities in the future? If yes, please explain.
13. Do your career aspirations include full- or part-time research in the future (i.e. in an academic medical post linked to a university?) If yes, please explain.
14. Please comment on how frequently you undertake the following activities as an undergraduate tutor (Options: Never, <1 time/month, 1-3 times/month, 1-3 times/week, >3 times/week):
    1. Delivering scheduled lectures
    2. Teaching small group or bedside tutorials
    3. Teaching online tutorials e.g. Zoom
    4. Writing teaching rosters
    5. Running SIM learning with students
    6. Organising and cooridinating teaching activities within my department
    7. Conducting lab-based research
    8. Conducting clinical research
    9. Meeting with supervisor(s) to discuss progress
    10. Carrying out clinical responsibilities during regular hours
    11. On-call work
    12. Assessing or contributing to the assessment of students
    13. Teaching other healthcare staff (nurses/physios, etc.)
15. For each of those activities, please comment about how important you think they are in your role as an undergraduate tutor/lecturer (Options: Not at all important, slightly important, moderately important, very important, extremely important).
    1. Delivering scheduled lectures
    2. Teaching small group or bedside tutorials
    3. Teaching online tutorials e.g. Zoom
    4. Writing teaching rosters
    5. Running SIM learning with students
    6. Organising and cooridinating teaching activities within my department
    7. Conducting lab-based research
    8. Conducting clinical research
    9. Meeting with supervisor(s) to discuss progress
    10. Carrying out clinical responsibilities during regular hours
    11. On-call work
    12. Assessing or contributing to the assessment of students
    13. Teaching other healthcare staff (nurses/physios, etc.)
16. In the tutor post, have you had work-related commitments which you feel are outside of your designated role? If yes, please explain.
17. Does your job include teaching qualified doctors? If yes, please explain.
18. How many hours approximately do you spend on site (in the hospital/university) each week teaching or preparing material relevant to your teaching? (Options <4 hours, 4-9 hours, 10-14 hours, >15 hours)
19. How many hours approximately do you spend off site (at home) doing education-related work each week? (Options <4 hours, 4-9 hours, 10-14 hours, >15 hours)
20. How many hours approximately do you spend each week doing clinical work? (Options <4 hours, 4-9 hours, 10-14 hours, >15 hours)
21. Is your teaching guided by a structured learning curriculum?
22. How does the reality of this job compare with your expectation of it? (e.g. more/less satisfying, easier/more difficult?) If it is very different, why is that?
23. Have you met any resistance or criticism from hospital or university staff about your teaching (the topics, the techniques, feedback)? If yes, please explain
24. Please comment below about how frequently you receive feedback on your role as the lecturer/tutor. (Options never, <1 time/month, 1-3 times/month, 1-3 times/week, >3 times/week):
25. Informal verbal feedback from clinical or university supervisor
26. Informal verbal feedback from students
27. Formal written feedback from students
28. Formal written feedback from clinical department or university
29. Are there any educational aspects of the job that you feel are beyond your experience and/or skill level? If yes, please explain.
30. Are there any aspects of the job that you feel should be done by other hospital and/or university staff? If yes, please explain.
31. For each of the following statements about the lecturer/tutor job, please select 1 option from 1-5 (Options: Strongly disagree, somewhat disagree, neither agree nor disagree, somewhat agree, strongly agree):
32. The job has improved my knowledge and/or skills in medical education
33. The job has improved my knowledge and/or skills in conducting lab-based research
34. The job has improved my knowledge and/or skills in conducting clinical research
35. The job has improved my knowledge and/or skills in the linked clinical specialty
36. This job has made me more likely to consider medical education as a major component of my future career
37. The job has provided me with knowledge and/or skills that will be useful for me during my career
38. What are the most enjoyable or satisfying aspects of the job?
39. What are the most difficult or challenging aspects of the job?
40. Would you consider re-applying for the same post?
41. Have you any other comments you would like to share with us about your experience of the lecturer/tutor role?

SEMI-STRUCTURED INTERIEWS

1. Tell me about the strengths and weaknesses of the post.
2. Do you think you successfully balanced the 3 roles of teacher, doctor and researcher? Can you tell me a bit about that?
3. What do you think are the most useful activities you do to help students learn?
4. The survey suggests that formal education training *for* the tutors in the post is uncommon. What training do you think would be most beneficial?
5. The survey suggests that the posts are often self-directed; that is the tutor makes their own decision about how to organise and deliver teaching. Was this your experience? Is this effective or could it be improved upon?
6. Survey respondents complained about the ill-defined nature of the job; a lack of clear "job description". What is your opinion on this? How would a clearer job description help with your job?
7. Is the job what you expected it to be? Is it better or worse than you expected? Why/why not?
8. The survey showed that very few tutors receive feedback on their student teaching work? Was this your experience? Do you think this has an impact on your job performance? Or perhaps on how effective your teaching has been?
9. The survey indicated that tutors spend a lot of time counselling/supporting students rather than teaching them. Was this your experience? What is your opinion about a tutor taking on this role?
10. We are interested in the perceived identity of tutors who responded to the survey. They described themselves variously as doctor, teacher, counsellor, motivator, administrator, researcher and general psychological support for students. Can you tell me the words you would use to best describe yourself in this job?
11. Do you ever experience any conflict between the expectations of your clinical department and administrative/SOM colleagues/students?
12. So in summary, what do you think would improve the job and make your educational role more effective?
13. Is there anything else you would like to add about the job, anything you think is relevant? Anything that you think might improve it?
14. Do you think that your clinical NCHD colleagues have a good understanding of what your job entails?

**APPENDIX 2: Cronbach Alpha of Internal Consistency:**

| Frequency of individual jobs | *0.58* |
| --- | --- |
| Importance of individual jobs | *0.78* |
| Time spent doing different types of work(on-site, off-site, clinical work) | *0.45* |
| Frequency of different types of feedback | *0.73* |
| The job has improved my knowledge | *0.90* |
